# Early Life Stress Differentially Modulates Distinct Forms of Brain Plasticity in Young and Adult Mice

**DOI:** 10.1371/journal.pone.0046004

**Published:** 2012-10-05

**Authors:** Inga Herpfer, Henning Hezel, Wilfried Reichardt, Kristin Clark, Julia Geiger, Claus M. Gross, Andrea Heyer, Valentin Neagu, Harsharan Bhatia, Hasan C. Atas, Bernd L. Fiebich, Josef Bischofberger, Carola A. Haas, Klaus Lieb, Claus Normann

**Affiliations:** 1 Department of Psychiatry and Psychotherapy, University of Freiburg Medical School, Freiburg, Germany; 2 Department of Radiology, Medical Physics, University of Freiburg Medical School, Freiburg, Germany; 3 Department of Biomedicine, University of Basel, Basel, Switzerland; 4 Experimental Epilepsy Group, Neurocenter, University of Freiburg Medical School, Freiburg, Germany; 5 Department of Psychiatry, University Medical Center Mainz, Mainz, Germany; University of Queensland, Australia

## Abstract

**Background:**

Early life trauma is an important risk factor for many psychiatric and somatic disorders in adulthood. As a growing body of evidence suggests that brain plasticity is disturbed in affective disorders, we examined the short-term and remote effects of early life stress on different forms of brain plasticity.

**Methodology/Principal Findings:**

Mice were subjected to early deprivation by individually separating pups from their dam in the first two weeks after birth. Distinct forms of brain plasticity were assessed in the hippocampus by longitudinal MR volumetry, immunohistochemistry of neurogenesis, and whole-cell patch-clamp measurements of synaptic plasticity. Depression-related behavior was assessed by the forced swimming test in adult animals. Neuropeptides and their receptors were determined by real-time PCR and immunoassay. Early maternal deprivation caused a loss of hippocampal volume, which returned to normal in adulthood. Adult neurogenesis was unaffected by early life stress. Long-term synaptic potentiation, however, was normal immediately after the end of the stress protocol but was impaired in adult animals. In the forced swimming test, adult animals that had been subjected to early life stress showed increased immobility time. Levels of substance P were increased both in young and adult animals after early deprivation.

**Conclusion:**

Hippocampal volume was affected by early life stress but recovered in adulthood which corresponded to normal adult neurogenesis. Synaptic plasticity, however, exhibited a delayed impairment. The modulation of synaptic plasticity by early life stress might contribute to affective dysfunction in adulthood.

## Introduction

Brain plasticity is highly sensitive to stress. Acute and chronic stressors downregulate both morphological and functional plasticity.

An important form of morphological brain plasticity is adult neurogenesis [1]. It has been shown in several species that the number of proliferating cells in the dentate gyrus diminishes shortly after acute and chronic stress [2], [3]. Moreover, other morphological changes in response to stress have been shown in the hippocampus, including retraction of apical dendrites of CA3 pyramidal neurons and loss of astrocytes [4], [5]. These findings might explain, at least in part, the hippocampal volume loss that has been observed after stress [6], [7]. In humans, there have been repeated reports of decreased hippocampal volume in stress-related disorders as depression and posttraumatic stress disorder [8], [9].

Whereas adult neurogenesis and stress-related brain volume loss are limited to distinct brain regions, plastic alterations of synaptic transmission are found ubiquitously in the brain. Long-term potentiation (LTP) increases synaptic efficacy, whereas long-term depression (LTD) decreases synaptic efficacy. As long-term synaptic plasticity can be measured for weeks or even months in living animals, it has been postulated that it is the molecular correlate of learning and memory [10], [11]. Stress has a profound impact on long-term synaptic plasticity. Both acute and chronic stress have been shown to facilitate hippocampal LTD and to impair LTP [12], [13].

Most of these findings were obtained directly after the end of the stress protocol, describing immediate effects of stress on brain plasticity. Much less is known about the long-term effects of stress. For example, little is known about how stress during early development modulates brain plasticity in adulthood. Remote effects of early life stress could be highly relevant; adverse childhood experiences that are associated with high levels of prolonged stress have been identified as important risk factors for a multitude of psychiatric and medical disorders, including depression, posttraumatic stress disorder, personality disorders, and cognitive dysfunction, as well as obesity, diabetes, and cardiovascular disease [14], [15], [16].

We applied a combination of in vivo and ex vivo methods to elucidate the effects of early life stress on different forms of plasticity in the hippocampus. We used the early deprivation (ED) paradigm, in which individual mouse pups were isolated from their dam and littermates repeatedly during the first two weeks of their postnatal development. In rats, ED has been shown to induce reduced body weight of pups and to modify the acoustical features of their ultrasonic vocalizations when separated from their dam. ED has also been found to affect maternal care upon retrieval of pups [17], [18]. In adult rats that had undergone ED, decreased emotionality in novel settings, a tendency for increased anxiety, anhedonia-like traits, and reduced social motivation were found [19], [20]. In addition, an ED-induced reduction in astroglial density was seen in different brain regions of adult rats [4]. In marmosets, ED has a long-term effect on the hippocampal expression of genes implicated in synaptic function and plasticity but does not affect hippocampal volume [21].

To assess immediate and long-term effects of ED on brain plasticity, we measured neurogenesis, hippocampal volume, long-term synaptic plasticity, stress-related behavior, neuropeptides and their receptors after termination of ED and in early adulthood. We found a clear dissociation between different forms of brain plasticity in response to early life stress.

## Results

C57/BL6 mice were individually separated from both their dam and littermates for three hours daily from postnatal day 1 (P1) to P14 and were compared to control animals that had been reared under routine animal facility conditions. Most of the measurements were performed at P15 and P70.

### Hippocampal volume

**Figure 1:**
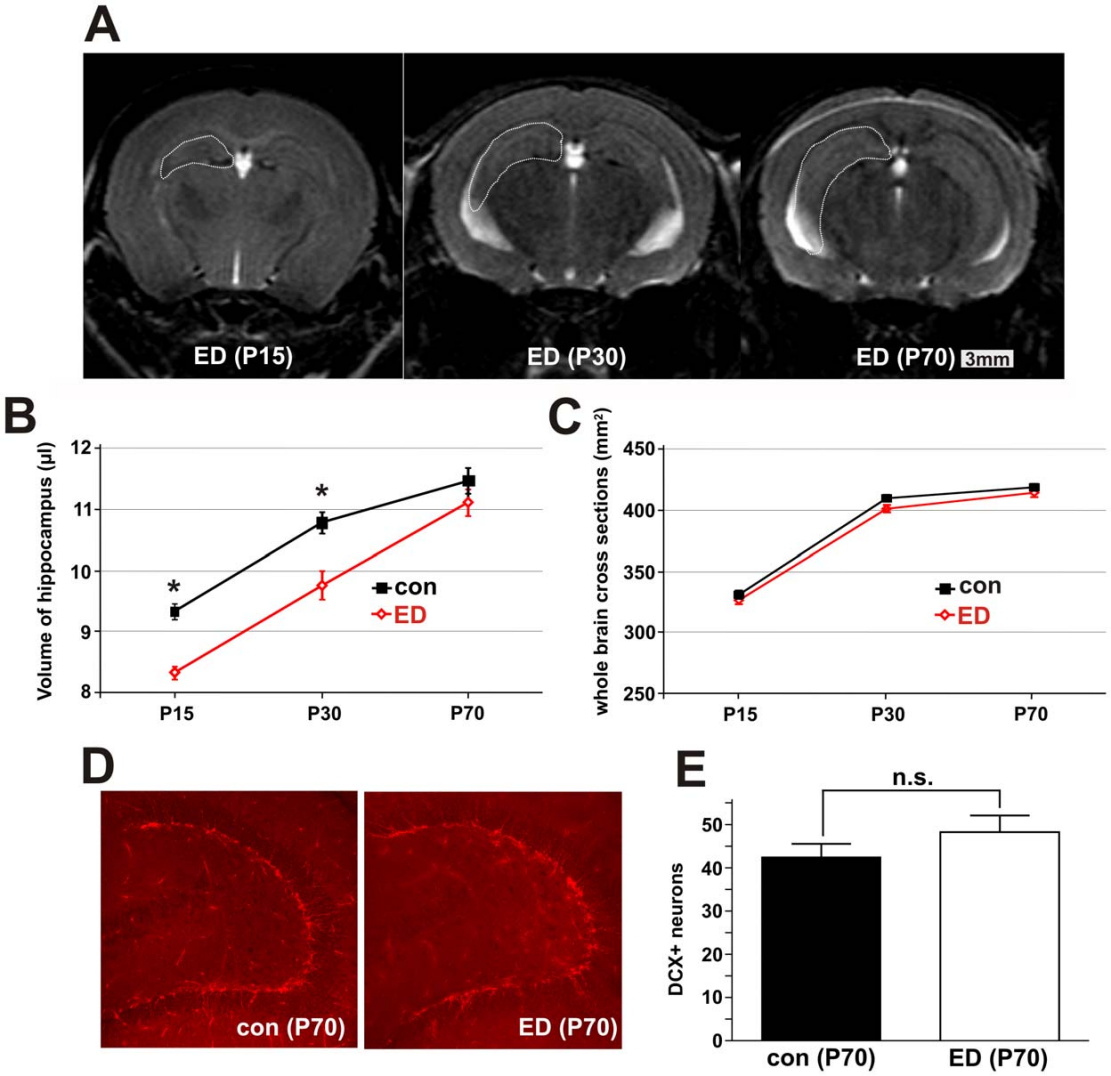
Reversible morphological alterations induced by early deprivation. *(A)* Hippocampus (dashed line) imaged using *in vivo* MRI in mice. T2-weighted MRI images taken longitudinally at P15, P30, and P70 *in vivo* (Scale bar: 3 mm). *(B)* Volumetric measurements of hippocampal volumes in different experimental groups. At P15 and P30, hippocampal volumes of ED group were significantly lower than in control mice, whereas at P70, there was no significant volumetric difference (n = 12 individual animals in each group; averages ± SEM). *(C)* Cumulative areas of 8 consecutive whole-brain cross-sections containing the hippocampus. There was no significant difference between deprived and non-deprived animals at either time point; arguing against an unspecific brain maturation deficit in deprived mice. *(D)* Doublecortin immunostaining of newborn neurons in the dentate gyrus of the P70 hippocampus. Projection images are from confocal stacks with a total thickness of 30 µM from control and ED animals. *(E)* No significant difference in neurogenesis between control and ED mice on day P70 (control: n = 11, ED: n = 9). Error bars represent SEM.

To assess morphological brain plasticity, we measured hippocampal volume longitudinally *in vivo* using magnetic resonance volumetry in individual animals at three different time points (P15, P30, and P70). We found that, at P15 and P30, hippocampal volumes of early deprived mice were significantly lower than those of control mice; however, at P70, no significant volumetric differences could be seen (Fig. 1 A and B). To control for an unspecific brain maturation deficit in deprived mice, we measured the cumulative areas of the consecutive whole-brain cross-sections containing the hippocampi and found no significant difference between stressed and non-stressed animals at all three time points (Fig. 1 C).

These results suggests that early deprivation induced specific and reversible morphological alterations in the hippocampus that normalized by adulthood.

### Hippocampal neurogenesis

Adult neurogenesis in the hippocampal dentate gyrus was assessed at P70 using immunohistochemistry for doublecortin. This protein is expressed selectively in newborn hippocampal granule cells during the first three weeks after mitosis and can therefore be used as a marker for adult neurogenesis. We found no significant difference in the number of newly generated neurons at P70 between ED and control animals (42.5±3.1 labeled cells in control animals, n = 11, vs. 48.2±3.9 cells in ED animals, n = 9, in the 30 µm projection image, n.s.; Fig. 1 D and E). We conclude that ED in early development is not sufficient to impair adult neurogenesis, corresponding with normal hippocampal volumes in adult animals from both experimental groups.

### Membrane and EPSP properties

**Figure 2:**
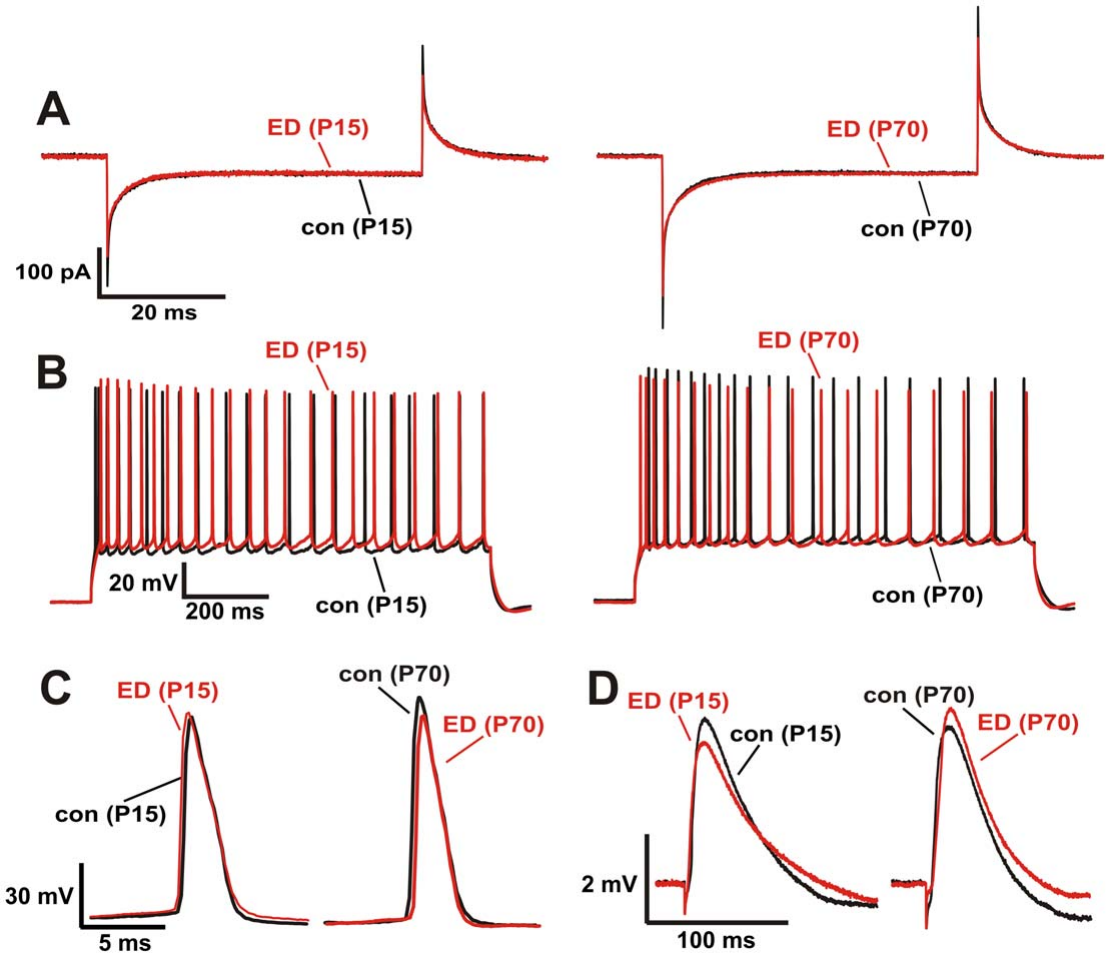
No alteration of membrane, AP and EPSP properties by early deprivation. Representative whole-cell measurements from single neurons in brain slices obtained from P15 (left) and P70 (right) mice after normal rearing conditions (black traces) and early deprivation (red traces). There were no significant differences in membrane, AP and EPSP properties between control animals and animals after early deprivation with the exception of a small but significant difference in the AP amplitude between the two experimental groups at P70 *(A)* Current response to a hyperpolarizing pulse of −5 mV from a membrane potential of −70 mV. Average of 10 consecutive traces from a single cell. *(B)* Train of action potentials evoked by a depolarizing current pulse. Representative single traces. *(C)* Representative action potentials averaged from 10 sweeps. *(D)* EPSPs evoked by Schaffer collateral stimulation; averaged from 10 EPSPs from one cell.

We collected brain slices from deprived and non-deprived animals after the termination of the early stress protocol on day P15 and in adulthood (P70) and performed whole-cell patch-clamp measurements of hippocampal CA1 pyramidal cells. We first determined basic intrinsic membrane and action potential (AP) properties (Fig. 2, Table 1). The membrane input resistance (R_M_) was calculated from the current response to a hyperpolarizing voltage pulse (−5 mV from a holding potential of −70 mV for 50 ms). There was no significant difference between R_M_ values in deprived or non-deprived mice at any time point (Fig. 2 A). Action potentials in CA1 neurons were evoked by a current injection of 300–800 pA for 1 s. Neither the AP half-width nor the frequency of the AP burst were different between the groups; however, there was a small but significant difference in the AP amplitude between the two experimental groups at P70 which could not be detected at P15 (Fig. 2 B, C). Excitatory postsynaptic potentials (EPSPs) were evoked by extracellular stimulation of the Schaffer collateral pathway. Neither EPSP rise time nor half width were significantly altered (Fig. 2 D). These findings argue against a functionally significant modulation of basic membrane conductances by the early deprivation protocol.

**Table 1:** Membrane Properties, Action Potential and Synaptic Function in the Hippocampal CA 1 Region of Juvenile and Adult Mice with and without Early Deprivation.

	Con (P15) (n)	ED (P15) (n)	Sign.	Con (P70) (n)	ED (P70) (n)	Sign.
**R_M_ (MΩ)**	193.2±10.1 (17)	188.4±13.1 (12)	ns	136.9±15.0 (9)	162.5±9.8 (11)	ns
**AP Amplitude (mV)**	82.1±1.1 (18)	83.1±1.7 (12)	ns	91.9±1.3 (9)	85.8±1.4 (11)	p<.006
**AP Half Width (msec)**	1.29±.06 (18)	1.19±.06 (12)	ns	1.02±.04 (9)	1.11±.05 (11)	ns
**AP Frequency (Hz)**	19.9±1.2 (18)	20.4±3.4 (12)	ns	19.0±1.8 (9)	15.8±1.8 (11)	ns
**EPSP Rise Time (msec)**	5.9±.3 (18)	5.3±.2 (12)	ns	5.1±.3 (9)	5.3±.3 (11)	ns
**EPSP Half Width (msec)**	32.2±2.2 (21)	28.1±1.1 (12)	ns	28.6±1.0 (9)	27.2±1.2 (11)	ns

R_M_, membrane input resistance; ns, nonsignificant; AP, action potential; EPSP, excitatory postsynaptic potential.

### Long-term synaptic plasticity

Our results so far suggested that ED impaired hippocampal volume in infant and juvenile mice but that these alterations recover by adulthood. We proceeded to examine putative effects of ED on long-term synaptic plasticity.

**Figure 3:**
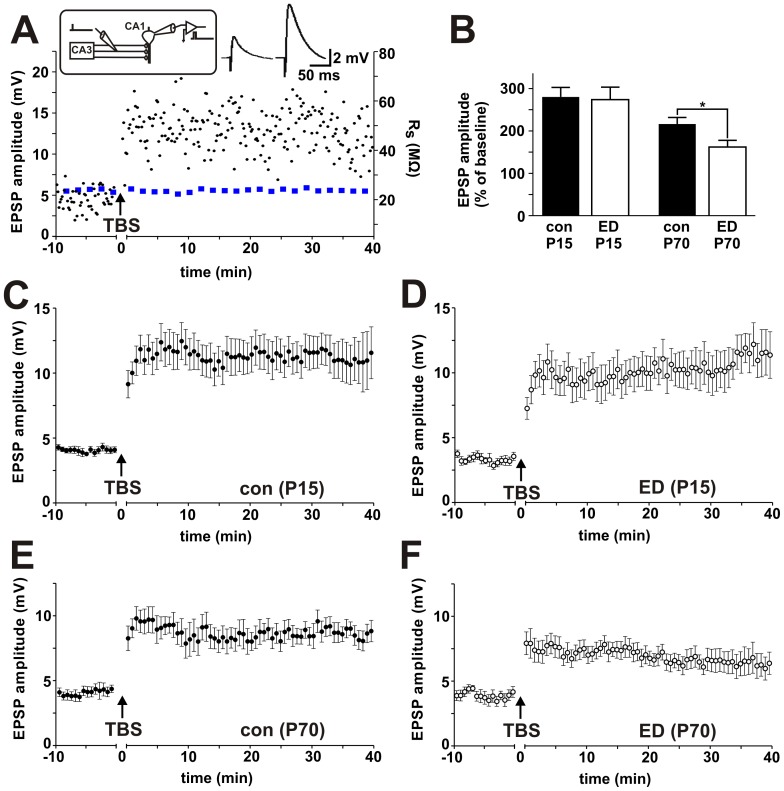
Theta-burst LTP is impaired in adult animals after early deprivation. *(A)* Whole-cell patch clamp recordings were made from CA1 pyramidal neurons in hippocampal brain slices. EPSPs were induced by current stimulation of the Schaffer collateral pathway. A typical single experiment from a non-stressed mouse is shown. Dots represent maximal EPSP amplitudes (left axis). Blue squares indicate series resistance (Rs, MΩ, right axis). At the time indicated by an arrow, LTP was induced by theta-burst stimulation (TBS), with 125 EPSP-AP pairings. This resulted in stable LTP. *(B)* Averaged effect of TBS-LTP in different experimental groups. Effect of TBS-LTP in control animals at P15 (n = 18, *C*); after ED at P15 (n = 12, *D*); in control animals at P70 (n = 9, *E*); and after ED at P70 (n = 11, *F*). All error bars represent SEM.

LTP was evoked using an associative, spike time-dependent, theta burst stimulation (TBS) protocol using a total of 125 EPSP - action potential (AP) pairings [22]. Five EPSPs were paired with five postsynaptic action potentials at a frequency of 50 Hz (delay between onset of EPSP and AP onset, 5 ms). Five of these pairing sequences were repeated at 5 Hz and five of the resulting theta-burst blocks were repeated at a frequency of 0.1 Hz (Fig. 3 A, B). Baseline EPSP amplitudes were not significantly different between the groups. The effect of the stimulation protocol within one experimental group was assessed by comparing baseline and post-stimulation EPSP amplitudes by a Wilcoxon signed rank test. Differences in the amount of LTP between the control and ED groups were compared by a Mann-Whitney test at P15 and P70.

In non-stressed animals at P15, the stimulation protocol resulted in a stable potentiation of synaptic transmission to 280.2±22.5% of baseline EPSP amplitude (n = 18, p<0.001 vs. baseline; Fig. 3 C). When the same protocol was applied to animals after the termination of ED at P15, the amount of potentiation was similar (273.4±29.6% of baseline EPSP amplitude, n = 12, p<0.001; Fig. 3 D). At P15, the amount of LTP was not significantly different between the control and the ED group.

When LTP was induced in non-stressed control animals at P70, an identical TBS protocol caused a potentiation to 216.4%±15.5% of baseline EPSP amplitude (n = 9, p<0.001 va. baseline; Fig. 3 E). However, in animals that had been subjected to ED in their postnatal period, tetanic stimulation resulted in a reduced amount of potentiation (161.9%±15.8% of baseline EPSP amplitude, n = 11, p<0.001 vs. baseline; Fig. 3 F). At P70, the amount of LTP was significantly lower in the ED group (p<0.05). Taken together, these results show that ED had no acute effect on LTP immediately after the end of the stress protocol. However, we noted a delayed impairment of long-term synaptic potentiation in adult animals after ED.

### Forced swimming test

In the forced swimming test (FST), rodents are placed in a small water basin; immobility time (floating) is compared to time spent with active escape movements (struggling). The FST tests the behavioral consequences of a short episode of stress. Many antidepressants selectively reduce floating time in the FST. 

**Figure 4:**
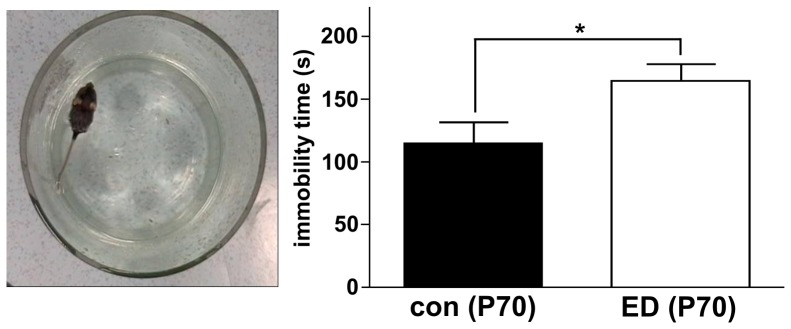
Increased immobility in the forced swimming test after early deprivation. Mice were placed in a water basin at P70 and behavior was videotaped. Immobility time represents the cumulative time spend with passive floating from minute 2 to 6 after the start of the experiment. Immobility time was significantly increased in adult mice which had been deprived after birth (n = 9 each).

The FST was performed in animals at P70. Previous exposure to ED significantly increased floating time (165.0±12.3 sec in ED mice vs. 114.9±17.0 sec in control animals, n = 9 each, p<0.05; Fig. 4). These findings suggest that postnatal exposure to ED has the opposite effect of antidepressant in the FST in adult mice.

### Neuropeptides and their receptors

**Figure 5:**
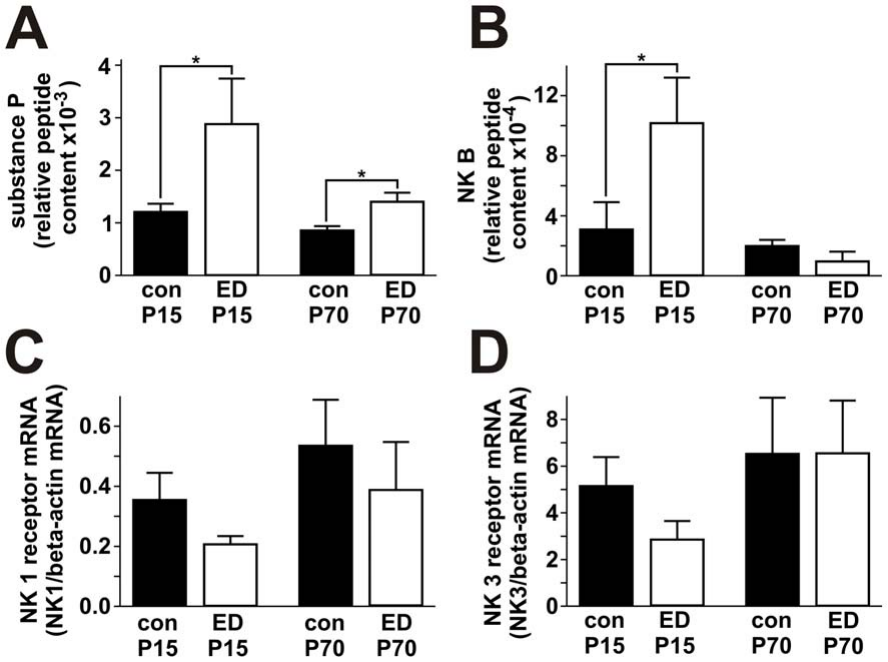
Neurochemical alterations induced by early deprivation. At P15, the content of substance P *(A)* and neurokinin B *(B)* in the frontal cortex of early deprived mice is significantly elevated compared to control animals. For substance P, this difference remains significant at P70, whereas neurokinin B levels return to normal. The differences in NK1 *(C)* and NK3 *(D)* receptor mRNA were not significant. All error bars represent SEM.

It is unclear how early life stress causes prolonged or delayed plastic alterations in adult life. Substance P and neurokinin B are two neuropeptides that are increased in different brain regions after exposure of animals to stress [23]. We quantified the cerebral concentrations of substance P and neurokinin B and their respective receptors NK-1 and NK-3 (Table 2). We found that, at P15, early deprivation was associated with significantly increased synthesis of substance P and neurokinin B in the frontal cortex, as measured by enzyme immunoassay. Substance P remained significantly increased until P70 (Fig. 5 A and B). At the same time, the synthesis of NK-1 and NK-3 receptors was downregulated as measured by mRNA real-time PCR; however, the decreased receptor mRNA concentration did not reach significance (Fig. 5 C and D). These results confirm that ED acts as an effective stressor in newborn mice and activates stress related pathways. Moreover, permanently increased substance P levels might suggest an involvement of tachykinine signaling in adult life alterations in response to early stress.

**Table 2:** Neuropeptides and their receptors.

	Con (P15) (n)	ED (P15) (n)	Sign.	Con (P70) (n)	ED (P70) (n)	Sign.
**Substance P**	1.19±.16 (6)	2.88±.87 (5)	p<.05	0.85±.08 (5)	1.53±.13 (5)	p<.05
**Neurokinin B**	3.05±.18 (6)	10.14±.30 (5)	p<.05	1.95±.04 (5)	0.93±.06 (6)	ns
**NK 1 receptor mRNA**	0.35±.09 (5)	0.21±.03 (5)	ns	0.53±.15 (5)	0.39±.15 (6)	ns
**NK 3 receptor mRNA**	5.13±1.2 (5)	2.83±.79 (4)	ns	6.51±1.2 (5)	6.53±2.26 (4)	ns

Concentrations of substance P and neurokinin B protein and NK 1 and NK 2 receptor mRNA in the frontal cortex at different time points after early deprivation and under control conditions.

Units: Substance P, relative peptide content ×10^−3^; Neurokinin B, relative peptide content ×10^−4^; NK 1 and NK 3 receptor mRNA, relation to beta-actin mRNA. ns, non-significant.

## Discussion

In sum, we found that ED was associated with reduced hippocampal volume in infant and juvenile mice; however, these alterations were corrected by adulthood. Neurogenesis in the dentate gyrus of adult mice after ED was unchanged. Early life stress caused a delayed impairment of LTP and increased immobility in the FST. Moreover, ED was associated with a sustained increased in the synthesis of substance P (Table 3). Taken together, these results suggest a dissociation of the effects of early life stress on different forms of brain plasticity. Whereas ED impairs hippocampal volume as an important measure of morphological plasticity in infant and juvenile mice, it causes delayed impairment of synaptic plasticity in adult animals.

**Table 3:** Main effects of early deprivation.

	ED vs control P15	ED vs. control P70
Hippocampal volume	↓	↔
Adult neurogenesis		↔
LTP	↔	↓
Immobility in FST		↑
Substance P	↑	↑

↓, significant decrease; ↑, significant increase; ↔, no significant difference.

### Reversible impairments of morphological brain plasticity after early life stress

A volumetric approach using MR imaging has been applied in a number of recent animal studies on rodents [24], [25], [26], [27], promoted by the increasing availability of dedicated animal imaging systems. Non-invasive in vivo measurements are especially useful because longitudinal studies offer more reliable measurements when they are repeated on the same animals over a longer period of time [28].

Despite its obvious advantages, volumetric measurements might not be able to detect small effects on hippocampal volume; moreover, it cannot be ruled out by volumetric assessments that early life stress is associated with long-lasting changes in other morphological measures like dendritic arborization, spine density, and spine morphology. Given the modulation of long-term synaptic plasticity by early life stress shown here, it is even highly likely that associated structural changes occur as LTP has been demonstrated to modify the morphology of dendritic spines [29], [30].

Hippocampal volumes have not been studied in rodents following ED. Kalish et al. [25] reported reduced hippocampal volume in a rat model of trait anxiety. However, Schubert et al. did not find alterations in hippocampal volume in rats following seven weeks of social isolation after weaning [27].

In humans, MR volumetry has revealed a number of structural abnormalities in a variety of psychiatric disorders, including post-traumatic stress disorder [9], borderline personality disorder [31] and first-episode schizophrenia [32]; however, many of these results have been difficult to replicate. In major depression, the reductions in hippocampal volume were small and heterogeneous but overall significant in a meta-analysis. They correlated with chronicity of depression (i.e., total lifetime number of affective episodes) but not with mood state [33]. 

The normalization of hippocampal volumes after ED corresponds to our finding of unimpaired neurogenesis in adult mice. Similar results have been reported for maternal separation in rats in which normal numbers of BrdU-labeled cells were observed when measured 2–3 weeks after BrdU injection [34], [35]. Adult hippocampal neurogenesis has been implicated in the pathophysiology of depression and the behavioral effects of antidepressants [36]. Our findings argue against persistent or delayed alterations of hippocampal volume or adult neurogenesis after early life stress.

### Modulation of synaptic plasticity by acute, chronic and early life stress

The modulation of long-term synaptic plasticity by stress has been demonstrated in numerous behavioral models. Acute stress protocols have been found to facilitate LTD and to impair the induction of LTP [13], [37], [38]. These effects were generally dependent on the activation of glucocorticoid receptors [39]. Chronic stress produces comparable effects on synaptic plasticity [12]. In previous work, we have shown that chronic mild stress in young adult rats upregulated subsequent LTD induction, an effect that could be prevented by treatment with an antidepressant [3]. The effects of chronic stress have typically been independent of increased basal glucocorticoid levels. Accordingly, in parallel experiments using the identical ED procedure, the glucocorticoid receptor mRNA levels were not altered at P15 [40]. Acutely increased glucocorticoid levels might cause secondary effects and affections of the HPA axis might be more readily detectable by functional testing [41]. 

In our experiments, we show long-term, remote effects of early life stress on synaptic plasticity in adult animals. Correspondingly, Brunson et al. found impaired LTP in middle aged rats, but not in young rats, after fragmented maternal care during the early postnatal period. This impaired LTP was accompanied by a deterioration of complex cognitive behavior [42]. A similar early-life stress protocol led to decreased LTP at P53–57 and deficits in spatial learning. This effect could be overcome by exposure to an enriched environment, which was applied following the stress procedure [43]. Another group described a delayed impairment of the reinforcement of LTP after postnatal maternal separation [44]. Moreover, maternal separation had a delayed effect on hippocampal synaptic development as assessed by synaptophysin immunoreactivity [45]. A putative modulation of LTD by early deprivation is unknown and has not been the focus of this study.

### Mechanisms of modulation of synaptic plasticity by early life stress

The cellular mechanisms underlying delayed modulation of synaptic plasticity by early life stress are unclear. Our data argue against a modulation of basic membrane conductances or excitability by the early stress protocol. More relevant mechanisms might include alterations in gene expression or second messenger cascades probably caused by initial glucocorticoid signals. An important candidate that may explain the mediation of hippocampal plasticity by early life stress is CRH [46], [47], [48]. This hypothesis is supported by the finding that the effects of early life stress on cognitive function in middle-aged rats could be reproduced by administration of CRH into the immature rat brain [49]. Alternatively, a reduction of astrocytes might impair later LTP induction; this phenomenon has been observed after early deprivation in Fischer rats [4], [50]. 

In this study, we found a prolonged increase of frontal Substance P concentrations and a transient increase of NK B concentrations after ED. NK 1 and NK 3 receptor mRNA levels were downregulated accordingly, but these reductions did not reach significance which is most probably due to the underpowered sample size in this set of experiments. Substance P has been shown to be involved in regulation of brain plasticity. In NK1 receptor knockout mice, neurogenesis in the dentate gyrus and BDNF secretion were increased, while immobility time in the FST was reduced [51]. This finding has also been demonstrated in TAC1 mutant mice, which lack substance P, and in wild-type mice after application of a NK1 receptor antagonist [52], [53]. An NK1 receptor antagonist could prevent stress-induced reductions in neurogenesis and hippocampal volume [7]. Mechanisms by which substance P could impair LTP might be in an increase in GABAergic inhibition or intracellular IP_3_ levels [54], [55], [56]; but see [57]. 

Different forms of stress have been shown to affect substance P secretion [23]. Substance P levels were increased after chronic mild stress, an animal model of depression [58]. Acute or chronic stress protocols caused a downregulation of NK1 receptors and increased substance P release in the amygdala [59], [60]. 

In humans, intravenous infusion of substance P lowered mood and memory, whereas increased substance P levels could be measured in major depression and post-traumatic stress disorder. NK1 receptor antagonists have been evaluated as antidepressants [23], [61].

Taken together, this evidence suggests that substance P may be another attractive candidate that could explain the modulation of brain plasticity by early stress. However, rather than a simple correlation between substance P levels and plasticity measures, plasticity and behavior might be modulated by a complex interaction between neurokinins and such interrelated signaling systems as BDNF, CRH and monoamines. A putative etiological role for substance P after early stress can only be assessed by interventional studies using NK1 antagonists or with knockout mice, which will be the focus of future studies.

### Behavioral impairment in adulthood after early life stress

What are the functional and behavioral consequences of impaired synaptic plasticity after early life stress?

Synaptic long-term plasticity is believed to be the molecular basis of learning and memory. Alterations of synaptic plasticity might explain learning defects in animal models of early stress [42], [62]. Similar cognitive dysfunctions have been described after early stress in primates and humans [63], [64].

Moreover, it has been proposed that synaptic plasticity is involved in emotional regulation and that synaptic plasticity or its modulation might be disturbed in depressed patients [65], [66]. We have observed facilitation of LTD after chronic mild stress, a valid animal model of depression [3]. In adult rodents, the antidepressant fluoxetine facilitated ocular dominance plasticity, a form of plasticity in the visual cortex [67]. In addition, different antidepressants, the mood stabilizer lithium and electroconvulsive therapy have been shown to effectively modulate synaptic plasticity in different brain regions including the hippocampus [68], [69], [70], [71]. In depressed humans, plastic modulation of visually evoked potentials was altered, while chronic application of an antidepressant in healthy humans greatly enhanced plasticity [72]. Different learning paradigms which were used as behavioral correlates of plasticity in humans were altered in major depression [73].

In this set of experiments, we show that an impairment of LTP due to early life stress occurs alongside an increased floating time in the FST [74], [75]. The FST is widely used as a screening test for antidepressive properties of pharmacological substances but cannot be regarded as an animal model of depression [76]. Recently, efforts have been made to separate its stress-inducing properties (forced swimming) from its potential utility as a read-out of the behavioral state of the animal (immobility time) [77]. In any case, under our conditions ED had an opposite effect compared to antidepressant in adult animals. However, further research is necessary to clarify how impairments of synaptic plasticity and increased immobility on the FST are related and how they relate to the risk for depression.

Previous findings on affective and fear-related behaviors after early trauma in mice are contradictory: increased immobility time in the FST has been observed after maternal separation, which could be prevented by treatment with an antidepressant or with a glucocorticoid receptor antagonist [78], [79]. Anxiety and fear-related behaviors were differentially modulated in adult mice after maternal separation depending on sex and estrous cycle of the animal [80]. However, Millstein & Holmes [81] did not observe any consistent effect of an ED procedure on different anxiety- and depression-related phenotype in several mouse strains. As we left the pups at room temperature without additional warming during the ED procedure, this additional stressor might explain the positive findings under our conditions.

### Pathophysiological implications

In humans early life trauma is one of the most important risk factors for psychiatric disorders, most notably depression. Different early life traumatic events have been shown to increase the risk for depression in adults. These traumatic events include birth complications, physical, sexual, and emotional abuse and severe somatic disease [82]. In post-traumatic stress disorder, severe acute or chronic traumatic events cause deficits in memory functions [9] but also predispose patients to depression. An impairment of synaptic plasticity in adulthood caused by early life stress might predispose susceptible individuals to exhibit increased sensitivity of brain plasticity to stress, which might impair the function of neuronal circuits involved in mood regulation, causing the phenotype of affective disorders.

## Materials and Methods

### Animals

C57/BL6 mice were kept with *ad libitum* access to food and water. Animals were maintained in a temperature-controlled room with a light/dark cycle of 12∶12 h (lights on at 07:00 h). Experiments were performed during the light period of the cycle at 21°C and were conducted in accordance with the principles and procedures of local authorities for the Care and Use of Laboratory Animals (Regierungspräsidium Freiburg, permit numbers G-04/65, G-05/48, G-08/35, T-06/01).

### Early deprivation stress

Each litter of newborn mouse pups was randomly assigned to an experimental group at P1. We compared two experimental groups: control (standard animal facility rearing) and ED, where all pups of a litter were simultaneously separated from their dam. All pups were individually isolated in a small plastic container lined with bedding (10×15×10 cm) for three hours daily in the evening in a separate room within the same animal facility. ED was performed at room temperature. After separation, the pups were returned to their home cages, where they were reunited with the dam. ED was performed from P1 to P14. All animals were weaned at P21. Not more than three animals were used from a single litter for each experimental protocol. With the exception of the longitudinal MR measurements, no individual animal was used for more than one experiment. Both sexes were used. Two time points were chosen for the described analyses: P15 (immediate effect of ED) and P70 (long-term effect of ED). For MRI studies, one additional time point was chosen: P30 (medium-term effect of ED).

### MR volumetry

MRI measurements of hippocampal volume were obtained *in-vivo* using a 9.4 Tesla animal scanner (BioSpec 94/20, Bruker BioSpin) in anesthetized mice under spontaneous breathing conditions using isoflurane. The respiration rate was continuously monitored and gating was used to reduce movement artifacts during the scan. The MRI protocol consisted of a localizer sequence to plan the brain imaging; a T2-weighted RARE Sequence (TEeff 60 ms, slice thickness: 0.4 mm, MTX 196×196, FOV 18×18 mm^2^) was used to depict the hippocampus in mouse brain images. Total hippocampal volumes were calculated from sets of contiguous images by summing products of area measurements and slice thickness using MIPAV, a freely available medical image processing software package from the National Institutes of Health (Bethesda, USA). Additionally, the areas of 8 cross-sections from contiguous sets of images of the complete brain of each mouse were measured; starting with the slice where the frontal end of the hippocampus was detected. Data were analyzed with two-way ANOVA using ‘age’ and ‘treatment’ as main factors. Next, one-way ANOVA with ‘group’ as the factor was carried out, followed by the Holm-Sidak *post hoc* test (Sigma Stat 3.5).

### Electrophysiology

Synaptic plasticity was measured at P15 and P70 using whole-cell patch clamp experiments on hippocampal brain slices. Mice were anesthetized with isoflurane and were killed by decapitation, in accordance with governmental and institutional guidelines. Transverse brain slices (300 µm) were cut from the hippocampus with a vibratome (DTK-1000, Dosaka). Slices were incubated at 35°C for 20 min and were then stored at room temperature in a saline solution containing (in mM): 125 NaCl, 25 glucose, 25 NaHCO_3_, 1.25 NaH_2_PO_4_, 2.5 KCl, 2 CaCl_2_, 1 MgCl_2_ (equilibrated with 95%O_2_/5% CO_2_). In the recording chamber, slices were superfused at a flow rate of 5 ml/min with saline containing 20 µM picrotoxin to pharmacologically isolate excitatory transmission. Infrared differential contrast video microscopy was used (Axioskop 2 FS plus, Zeiss, IMAGO-VGA, Till Photonics). CA1 pyramidal neurons were identified visually and by their characteristic firing pattern. Patch pipettes were pulled from borosilicate glass tubing (3–6 MΩ, Hilgenberg). The recording pipette contained a filtered solution with (in mM): 135 K-gluconate, 20 KCl, 2 MgCl_2_, 4 sodium adenosine triphosphate, 0.3 sodium guanosine 5′-triphosphate, 10 HEPES, and 0.1 EGTA, equilibrated with KOH to pH 7.3. Whole cell recordings were made with an EPC-9 amplifier (HEKA). Signals were filtered at 5 and 10 kHz and were stored offline for later analysis using Pulse and Pulse Fit software (HEKA). All chemicals were obtained from Sigma-Aldrich, Merck or Tocris.

Schaffer collateral fibres were stimulated with a stimulus isolator (Stimulator 2100, A-M Systems) via a patch pipette that was filled with a HEPES-buffered NaCl solution (1–2 MΩ). The pipette was placed in stratum radiatum of the CA1 region, 20–50 µm away from the pyramidal cell layer. 100 µs voltage pulses of 10–100 V were applied at a frequency of 0.1 Hz to evoke an initial subthreshold EPSP amplitude of 3–6 mV. Before initiation of the induction protocol, a stable baseline was recorded for 10 min.

The resting membrane potential of most cells was between −68 and −72 mV; holding potential was set to −70 mV. Input and series resistances were continuously monitored and recorded over the course of the experiment by applying a 500 ms current pulse after every 10^th^ EPSP. The criteria for discarding an experiment were as follows: unstable baseline (>20% difference of EPSP amplitudes between −10 min and start of the stimulation), unstable EPSP amplitude after stimulation (>20% difference between 10 and 20 min after termination of the stimulation protocol), occurrence of sudden EPSP amplitude steps or distinct amplitude oscillations, change of >25% in series resistance during the course of the experiment, and evidence of ictal discharge or spiking. In addition, only experiments in which the LTP stimulation protocol caused a significant increase in EPSP amplitude were included into the final analysis (as calculated with a Wilcoxon signed rank test between the last 60 EPSP amplitudes before the stimulation and the 60 EPSP amplitudes between 20 and 30 min after stimulation). The number of excluded recordings was comparable between the different experimental series. Experiments examining the different groups were interleaved.

To quantify synaptic plasticity, 60 consecutive EPSPs were averaged 10 min before the initiation and 20–30 min after the termination of the induction protocol. Changes of the mean EPSP amplitude pre- and post-induction within an experimental series were assessed by a Wilcoxon signed rank test. Normalized plasticity measurements were compared between separate series using a Mann-Whitney test. GraphPad Prism software was used to average and plot EPSP amplitudes.

### Immunohistochemistry and imaging of neurogenesis

For doublecortin immunohistochemistry, 350 µm thick slices (one slice per animal) were used, with the same preparation technique as that of brain slices for patch clamp recording. Slices were fixed in 4% paraformaldehyde and were washed. After incubation overnight at room temperature with the primary antibody goat-anti-doublecortin (C-18, immunoglobulin G, 1∶100; together with 5% donkey serum and 0.3% Triton X-100, Santa Cruz Biotechnology), slices were washed three times for 30 min with 0.1 mol/l phosphate buffered saline. The secondary antibody donkey-anti-goat-Alexa 568 (1∶200, Invitrogen) was co-applied with 0.3% Triton X-100 for 24 hours at 4°C. After rinsing, slices were embedded in ProLong Antifade (Invitrogen). Immunofluorescence was measured with a confocal laser-scanning microscope (LSM 510, Zeiss). A projection image was constructed from confocal stacks with a total thickness of 30 µm. Stained cells were manually counted in the granule cell layer and in the subgranular zone of the dentate gyrus by two blinded investigators. Values were compared with the Mann-Whitney test (GraphPad Prism).

### Forced swimming test

In a calm room, mice were gently placed for 6 min in a cylindrical glass container (volume 3 l, diameter 19 cm) that was filled to a depth of 15 cm with water at a temperature of 25°C. Water was changed after every experiment. Behavior was videotaped and analyzed offline from 2 to 6 min after the start of the test by two independent investigators who were blinded to the experimental group of the animals. *Floating* was defined as either being stationary, with only enough movements of the tail or the forepaws to keep the head above water, or as calm swimming. Calm swimming did not involve lifting the paws above the water surface, with the body oriented parallel to the sides of the cylinder. *Struggling* was defined as active pawing of the side of the cylinder, reminiscent of escape movements, with the head of the animal oriented toward the wall and with the body oriented perpendicularly to the side of the cylinder [74], [75], [83]. Data were compared using Mann-Whitney test. As the FST is neither meaningful nor defined for very immature animals, these experiments could only be performed at P15.

### RNA isolation and quantitative real-time PCR

RNA isolation and quantitative real-time PCR was used to quantify the expression of NK1- and NK3- receptor mRNA. Total RNA was extracted from mouse brain (frontal cortex) using the guanidine isothiocyanate method. The quantity of total RNA was determined by spectrophotometric measurement at 260 nm. Total RNA (1 µg) was subjected to reverse transcription using 1 µg of random hexamer oligonucleotides in a 15 µl reaction volume. A master mix consisting of 1× reaction buffer (50 mM Tris-HCl pH 8.3, 75 mM KCl, 3 mM MgCl_2_, and 10 mM DTT), 0.5 mM deoxynucleotide triphosphates (dNTP mix, InViTek), 1 U/µl recombinant RNasin (Promega) and 200 U of Moloney murine leukemia virus RT (M-MLV RT, Promega) was added to the samples to a final volume of 25 µl/sample. Real-time PCR analysis was performed using the Roche Light Cycler 2.0 and the Light Cycler FastStart DNA Master plus SYBR Green I Kit, as described in the reference procedure, using 0.5 µM of each forward and reverse primer, 11 µl PCR-grade H_2_O and 1 µl cDNA. Primer pairs were designed with the analysis software Primer 3. The sequences of the primer pair for NK1-receptor were 5′-GCCAGAACATCCCAACAGG-3′ (sense) and 5′- GGCGAAGGTACACACAACCA-3′ (antisense) (expected size: 223 bp). The sequences of the primer pair for NK3-receptor were 5′-CCAACTACTGCCGCTTCCA-3′ (sense) and 5′-GAAATGTTGCTTGGGACCTTCT-3′ (antisense) (expected size: 272 bp). The sequences of the primer pair for ß-actin were 5′-TCCCTGGAGAAGAGCTACGA-3′ (sense) and 5′-ATCTGCTGGAAGGTGGACAG-3′ (antisense) (expected size: 362 bp). ß-actin was used as the endogenous control for normalization of initial RNA levels. Control samples with no added cDNA were also included with each run to detect any possible contamination. Thermal cycling conditions were designed as follows: initial denaturation at 95°C for 600 s followed by 40 cycles of 95°C for 10 s, 60°C for 15 s, and 72°C for 10 s. Fluorescence was monitored during each annealing step. At the end of each PCR run, the data were automatically analyzed by the system and an amplification plot was generated for each cDNA sample.

### Enzyme Immunoassay

Enzyme Immunoassay (EIA) was used to quantify the concentration of substance P and neurokinin B in frontal cortex. Peptides/proteins were isolated as described above. Neurokinin B levels were determined using the Neurokinin B EIA kit provided by Phoenix Pharmaceuticals (Burlingame, CA, USA). Substance P levels were measured using the Substance P EIA kit provided by Assay Designs (Ann Arbor, Michigan, USA). Protein levels were related to the total protein content of the lysate determined by a BCA Protein Assay Kit (Thermo Fisher Scientific, Rockford, USA). Molecular biology data were compared using a Mann-Whitney test.

## References

[1] Gage FH (2000) Mammalian neural stem cells. Science 287: 1433–1438. 10688783 10.1126/science.287.5457.1433

[2] Gould E, Tanapat P, McEwen BS, Flugge G, Fuchs E (1998) Proliferation of granule cell precursors in the dentate gyrus of adult monkeys is diminished by stress. Proc Natl Acad Sci U S A 95: 3168–3171. 9501234 10.1073/pnas.95.6.3168 PMC19713

[3] Holderbach R, Clark K, Moreau JL, Bischofberger J, Normann C (2007) Enhanced long-term synaptic depression in an animal model of depression. Biol Psychiatry 62: 92–100. 17141742 10.1016/j.biopsych.2006.07.007

[4] Leventopoulos M, Ruedi-Bettschen D, Knuesel I, Feldon J, Pryce CR, et al (2007) Long-term effects of early life deprivation on brain glia in Fischer rats. Brain Res 1142: 119–126. 17306230 10.1016/j.brainres.2007.01.039

[5] Magarinos AM, McEwen BS, Flugge G, Fuchs E (1996) Chronic psychosocial stress causes apical dendritic atrophy of hippocampal CA3 pyramidal neurons in subordinate tree shrews. J Neurosci 16: 3534–3540. 8627386 10.1523/JNEUROSCI.16-10-03534.1996 PMC6579123

[6] McEwen BS (1999) Stress and hippocampal plasticity. Annu Rev Neurosci 22: 105–122. 10202533 10.1146/annurev.neuro.22.1.105

[7] van der Hart MG, Czeh B, de Biurrun G, Michaelis T, Watanabe T, et al (2002) Substance P receptor antagonist and clomipramine prevent stress-induced alterations in cerebral metabolites, cytogenesis in the dentate gyrus and hippocampal volume. Mol Psychiatry 7: 933–941. 12399945 10.1038/sj.mp.4001130

[8] Bremner JD, Narayan M, Anderson ER, Staib LH, Miller HL, et al (2000) Hippocampal volume reduction in major depression. Am J Psychiatry 157: 115–118. 10618023 10.1176/ajp.157.1.115

[9] Bremner JD, Randall P, Scott TM, Bronen RA, Seibyl JP, et al (1995) MRI-based measurement of hippocampal volume in patients with combat-related posttraumatic stress disorder. Am J Psychiatry 152: 973–981. 7793467 10.1176/ajp.152.7.973 PMC3233767

[10] Bliss TV, Collingridge GL (1993) A synaptic model of memory: long-term potentiation in the hippocampus. Nature 361: 31–39. 8421494 10.1038/361031a0

[11] Kandel ER (2001) The molecular biology of memory storage: a dialogue between genes and synapses. Science 294: 1030–1038. 11691980 10.1126/science.1067020

[12] Pavlides C, Nivon LG, McEwen BS (2002) Effects of chronic stress on hippocampal long-term potentiation. Hippocampus 12: 245–257. 12000121 10.1002/hipo.1116

[13] Xu L, Anwyl R, Rowan MJ (1997) Behavioural stress facilitates the induction of long-term depression in the hippocampus. Nature 387: 497–500. 9168111 10.1038/387497a0

[14] Felitti VJ, Anda RF, Nordenberg D, Williamson DF, Spitz AM, et al (1998) Relationship of childhood abuse and household dysfunction to many of the leading causes of death in adults. The Adverse Childhood Experiences (ACE) Study. Am J Prev Med 14: 245–258. 9635069 10.1016/s0749-3797(98)00017-8

[15] Heim C, Nemeroff CB (2001) The role of childhood trauma in the neurobiology of mood and anxiety disorders: preclinical and clinical studies. Biol Psychiatry 49: 1023–1039. 11430844 10.1016/s0006-3223(01)01157-x

[16] Zanarini MC, Williams AA, Lewis RE, Reich RB, Vera SC, et al (1997) Reported pathological childhood experiences associated with the development of borderline personality disorder. Am J Psychiatry 154: 1101–1106. 9247396 10.1176/ajp.154.8.1101

[17] Ruedi-Bettschen D, Feldon J, Pryce CR (2004) Circadian- and temperature-specific effects of early deprivation on rat maternal care and pup development: short-term markers for long-term effects? Dev Psychobiol 45: 59–71. 15340975 10.1002/dev.20014

[18] Zimmerberg B, Kim JH, Davidson AN, Rosenthal AJ (2003) Early deprivation alters the vocalization behavior of neonates directing maternal attention in a rat model of child neglect. Ann N Y Acad Sci 1008: 308–313. 14998903 10.1196/annals.1301.039

[19] Marmendal M, Eriksson CJ, Fahlke C (2006) Early deprivation increases exploration and locomotion in adult male Wistar offspring. Pharmacol Biochem Behav 85: 535–544. 17109940 10.1016/j.pbb.2006.10.005

[20] Rees SL, Steiner M, Fleming AS (2006) Early deprivation, but not maternal separation, attenuates rise in corticosterone levels after exposure to a novel environment in both juvenile and adult female rats. Behav Brain Res 175: 383–391. 17081629 10.1016/j.bbr.2006.09.013

[21] Law AJ, Pei Q, Walker M, Gordon-Andrews H, Weickert CS, et al (2009) Early parental deprivation in the marmoset monkey produces long-term changes in hippocampal expression of genes involved in synaptic plasticity and implicated in mood disorder. Neuropsychopharmacology 34: 1381–1394. 18615010 10.1038/npp.2008.106 PMC2669475

[22] Schmidt-Hieber C, Jonas P, Bischofberger J (2004) Enhanced synaptic plasticity in newly generated granule cells of the adult hippocampus. Nature 429: 184–187. 15107864 10.1038/nature02553

[23] Herpfer I, Lieb K (2005) Substance P receptor antagonists in psychiatry: rationale for development and therapeutic potential. CNS Drugs 19: 275–293. 15813642 10.2165/00023210-200519040-00001

[24] Cerqueira JJ, Catania C, Sotiropoulos I, Schubert M, Kalisch R, et al (2005) Corticosteroid status influences the volume of the rat cingulate cortex - a magnetic resonance imaging study. J Psychiatr Res 39: 451–460. 15992553 10.1016/j.jpsychires.2005.01.003

[25] Kalisch R, Schubert M, Jacob W, Kessler MS, Hemauer R, et al (2006) Anxiety and hippocampus volume in the rat. Neuropsychopharmacology 31: 925–932. 16192979 10.1038/sj.npp.1300910

[26] Schubert MI, Kalisch R, Sotiropoulos I, Catania C, Sousa N, et al (2008) Effects of altered corticosteroid milieu on rat hippocampal neurochemistry and structure–an in vivo magnetic resonance spectroscopy and imaging study. J Psychiatr Res 42: 902–912. 18177670 10.1016/j.jpsychires.2007.10.003

[27] Schubert MI, Porkess MV, Dashdorj N, Fone KC, Auer DP (2009) Effects of social isolation rearing on the limbic brain: a combined behavioral and magnetic resonance imaging volumetry study in rats. Neuroscience 159: 21–30. 19141315 10.1016/j.neuroscience.2008.12.019

[28] Geuze E, Vermetten E, Bremner JD (2005) MR-based in vivo hippocampal volumetrics: 1. Review of methodologies currently employed. Mol Psychiatry 10: 147–159. 15340353 10.1038/sj.mp.4001580

[29] Engert F, Bonhoeffer T (1999) Dendritic spine changes associated with hippocampal long-term synaptic plasticity. Nature 399: 66–70. 10331391 10.1038/19978

[30] Toni N, Buchs PA, Nikonenko I, Bron CR, Muller D (1999) LTP promotes formation of multiple spine synapses between a single axon terminal and a dendrite. Nature 402: 421–425. 10586883 10.1038/46574

[31] Driessen M, Herrmann J, Stahl K, Zwaan M, Meier S, et al (2000) Magnetic resonance imaging volumes of the hippocampus and the amygdala in women with borderline personality disorder and early traumatization. Arch Gen Psychiatry 57: 1115–1122. 11115325 10.1001/archpsyc.57.12.1115

[32] Velakoulis D, Wood SJ, Wong MT, McGorry PD, Yung A, et al (2006) Hippocampal and amygdala volumes according to psychosis stage and diagnosis: a magnetic resonance imaging study of chronic schizophrenia, first-episode psychosis, and ultra-high-risk individuals. Arch Gen Psychiatry 63: 139–149. 16461856 10.1001/archpsyc.63.2.139

[33] Videbech P, Ravnkilde B (2004) Hippocampal volume and depression: a meta-analysis of MRI studies. Am J Psychiatry 161: 1957–1966. 15514393 10.1176/appi.ajp.161.11.1957

[34] Greisen MH, Altar CA, Bolwig TG, Whitehead R, Wortwein G (2005) Increased adult hippocampal brain-derived neurotrophic factor and normal levels of neurogenesis in maternal separation rats. J Neurosci Res 79: 772–778. 15690366 10.1002/jnr.20418

[35] Mirescu C, Peters JD, Gould E (2004) Early life experience alters response of adult neurogenesis to stress. Nat Neurosci 7: 841–846. 15273691 10.1038/nn1290

[36] Sahay A, Hen R (2007) Adult hippocampal neurogenesis in depression. Nature neuroscience 10: 1110–1115. 17726477 10.1038/nn1969

[37] Kim JJ, Foy MR, Thompson RF (1996) Behavioral stress modifies hippocampal plasticity through N-methyl-D-aspartate receptor activation. Proc Natl Acad Sci U S A 93: 4750–4753. 8643474 10.1073/pnas.93.10.4750 PMC39350

[38] Shors TJ, Seib TB, Levine S, Thompson RF (1989) Inescapable versus escapable shock modulates long-term potentiation in the rat hippocampus. Science 244: 224–226. 2704997 10.1126/science.2704997

[39] Xu L, Holscher C, Anwyl R, Rowan MJ (1998) Glucocorticoid receptor and protein/RNA synthesis-dependent mechanisms underlie the control of synaptic plasticity by stress. Proc Natl Acad Sci U S A 95: 3204–3208. 9501241 10.1073/pnas.95.6.3204 PMC19720

[40] Gross CM, Flubacher A, Tinnes S, Heyer A, Scheller M, et al (2012) Early life stress stimulates hippocampal reelin gene expression in a sex-specific manner: Evidence for corticosterone-mediated action. Hippocampus 22: 409–420. 21136520 10.1002/hipo.20907

[41] Ladd CO, Huot RL, Thrivikraman KV, Nemeroff CB, Plotsky PM (2004) Long-term adaptations in glucocorticoid receptor and mineralocorticoid receptor mRNA and negative feedback on the hypothalamo-pituitary-adrenal axis following neonatal maternal separation. Biol Psychiatry 55: 367–375. 14960289 10.1016/j.biopsych.2003.10.007

[42] Brunson KL, Kramar E, Lin B, Chen Y, Colgin LL, et al (2005) Mechanisms of late-onset cognitive decline after early-life stress. J Neurosci 25: 9328–9338. 16221841 10.1523/JNEUROSCI.2281-05.2005 PMC3100717

[43] Cui M, Yang Y, Yang J, Zhang J, Han H, et al (2006) Enriched environment experience overcomes the memory deficits and depressive-like behavior induced by early life stress. Neurosci Lett 404: 208–212. 16790315 10.1016/j.neulet.2006.05.048

[44] Gruss M, Braun K, Frey JU, Korz V (2008) Maternal separation during a specific postnatal time window prevents reinforcement of hippocampal long-term potentiation in adolescent rats. Neuroscience 152: 1–7. 18255235 10.1016/j.neuroscience.2007.12.033

[45] Andersen SL, Teicher MH (2004) Delayed effects of early stress on hippocampal development. Neuropsychopharmacology 29: 1988–1993. 15316569 10.1038/sj.npp.1300528

[46] Chen Y, Bender RA, Frotscher M, Baram TZ (2001) Novel and transient populations of corticotropin-releasing hormone-expressing neurons in developing hippocampus suggest unique functional roles: a quantitative spatiotemporal analysis. J Neurosci 21: 7171–7181. 11549728 10.1523/JNEUROSCI.21-18-07171.2001 PMC3107537

[47] Heim C, Owens MJ, Plotsky PM, Nemeroff CB (1997) Persistent changes in corticotropin-releasing factor systems due to early life stress: relationship to the pathophysiology of major depression and post-traumatic stress disorder. Psychopharmacol Bull 33: 185–192. 9230630

[48] Ivy AS, Rex CS, Chen Y, Dube C, Maras PM, et al (2010) Hippocampal dysfunction and cognitive impairments provoked by chronic early-life stress involve excessive activation of CRH receptors. The Journal of neuroscience : the official journal of the Society for Neuroscience 30: 13005–13015. 20881118 10.1523/JNEUROSCI.1784-10.2010 PMC2991143

[49] Brunson KL, Eghbal-Ahmadi M, Bender R, Chen Y, Baram TZ (2001) Long-term, progressive hippocampal cell loss and dysfunction induced by early-life administration of corticotropin-releasing hormone reproduce the effects of early-life stress. Proc Natl Acad Sci U S A 98: 8856–8861. 11447269 10.1073/pnas.151224898 PMC37525

[50] Henneberger C, Papouin T, Oliet SH, Rusakov DA (2010) Long-term potentiation depends on release of D-serine from astrocytes. Nature 463: 232–236. 20075918 10.1038/nature08673 PMC2807667

[51] Morcuende S, Gadd CA, Peters M, Moss A, Harris EA, et al (2003) Increased neurogenesis and brain-derived neurotrophic factor in neurokinin-1 receptor gene knockout mice. Eur J Neurosci 18: 1828–1836. 14622216 10.1046/j.1460-9568.2003.02911.x

[52] Bilkei-Gorzo A, Racz I, Michel K, Zimmer A (2002) Diminished anxiety- and depression-related behaviors in mice with selective deletion of the Tac1 gene. J Neurosci 22: 10046–10052. 12427862 10.1523/JNEUROSCI.22-22-10046.2002 PMC6757849

[53] Dableh LJ, Yashpal K, Rochford J, Henry JL (2005) Antidepressant-like effects of neurokinin receptor antagonists in the forced swim test in the rat. Eur J Pharmacol 507: 99–105. 15659299 10.1016/j.ejphar.2004.11.024 PMC5127697

[54] Kouznetsova M, Nistri A (1998) Modulation by substance P of synaptic transmission in the mouse hippocampal slice. Eur J Neurosci 10: 3076–3084. 9786202 10.1046/j.1460-9568.1998.00318.x

[55] Mantyh PW, Pinnock RD, Downes CP, Goedert M, Hunt SP (1984) Correlation between inositol phospholipid hydrolysis and substance P receptors in rat CNS. Nature 309: 795–797. 6204206 10.1038/309795a0

[56] Taufiq AM, Fujii S, Yamazaki Y, Sasaki H, Kaneko K, et al (2005) Involvement of IP3 receptors in LTP and LTD induction in guinea pig hippocampal CA1 neurons. Learn Mem 12: 594–600. 16287718 10.1101/lm.17405 PMC1356177

[57] Langosch JM, Kupferschmid S, Heinen M, Walden J, Herpfer I, et al (2005) Effects of substance P and its antagonist L-733060 on long term potentiation in guinea pig hippocampal slices. Prog Neuropsychopharmacol Biol Psychiatry 29: 315–319. 15694240 10.1016/j.pnpbp.2004.11.017

[58] Sergeyev V, Fetissov S, Mathe AA, Jimenez PA, Bartfai T, et al (2005) Neuropeptide expression in rats exposed to chronic mild stresses. Psychopharmacology (Berl) 178: 115–124. 15719227 10.1007/s00213-004-2015-3

[59] Duric V, McCarson KE (2005) Hippocampal neurokinin-1 receptor and brain-derived neurotrophic factor gene expression is decreased in rat models of pain and stress. Neuroscience 133: 999–1006. 15964488 10.1016/j.neuroscience.2005.04.002

[60] Ebner K, Rupniak NM, Saria A, Singewald N (2004) Substance P in the medial amygdala: emotional stress-sensitive release and modulation of anxiety-related behavior in rats. Proc Natl Acad Sci U S A 101: 4280–4285. 15024126 10.1073/pnas.0400794101 PMC384732

[61] Kramer MS, Cutler N, Feighner J, Shrivastava R, Carman J, et al (1998) Distinct mechanism for antidepressant activity by blockade of central substance P receptors. Science 281: 1640–1645. 9733503 10.1126/science.281.5383.1640

[62] Lehmann J, Pryce CR, Bettschen D, Feldon J (1999) The maternal separation paradigm and adult emotionality and cognition in male and female Wistar rats. Pharmacol Biochem Behav 64: 705–715. 10593193 10.1016/s0091-3057(99)00150-1

[63] Sanchez MM, Hearn EF, Do D, Rilling JK, Herndon JG (1998) Differential rearing affects corpus callosum size and cognitive function of rhesus monkeys. Brain Res 812: 38–49. 9813233 10.1016/s0006-8993(98)00857-9

[64] Bhutta AT, Anand KJ (2001) Abnormal cognition and behavior in preterm neonates linked to smaller brain volumes. Trends Neurosci 24: 129–130; discussion 131–122. 11182440 10.1016/s0166-2236(00)01747-1

[65] Castren E (2005) Is mood chemistry? Nat Rev Neurosci 6: 241–246. 15738959 10.1038/nrn1629

[66] Spedding M, Neau I, Harsing L (2003) Brain plasticity and pathology in psychiatric disease: sites of action for potential therapy. Curr Opin Pharmacol 3: 33–40. 12550739 10.1016/s1471-4892(02)00008-5

[67] Maya Vetencourt JF, Sale A, Viegi A, Baroncelli L, De Pasquale R, et al (2008) The antidepressant fluoxetine restores plasticity in the adult visual cortex. Science 320: 385–388. 18420937 10.1126/science.1150516

[68] Levkovitz Y, Grisaru N, Segal M (2001) Transcranial magnetic stimulation and antidepressive drugs share similar cellular effects in rat hippocampus. Neuropsychopharmacology 24: 608–616. 11331140 10.1016/S0893-133X(00)00244-X

[69] Niehusmann P, Seifert G, Clark K, Atas HC, Herpfer I, et al (2010) Coincidence detection and stress modulation of spike time-dependent long-term depression in the hippocampus. J Neurosci 30: 6225–6235. 20445048 10.1523/JNEUROSCI.6411-09.2010 PMC6632706

[70] Von Frijtag JC, Kamal A, Reijmers LG, Schrama LH, van den Bos R, et al (2001) Chronic imipramine treatment partially reverses the long-term changes of hippocampal synaptic plasticity in socially stressed rats. Neurosci Lett 309: 153–156. 11514064 10.1016/s0304-3940(01)02062-6

[71] Karpova NN, Pickenhagen A, Lindholm J, Tiraboschi E, Kulesskaya N, et al (2011) Fear erasure in mice requires synergy between antidepressant drugs and extinction training. Science 334: 1731–1734. 22194582 10.1126/science.1214592 PMC3929964

[72] Normann C, Schmitz D, Furmaier A, Doing C, Bach M (2007) Long-term plasticity of visually evoked potentials in humans is altered in major depression. Biol Psychiatry 62: 373–380. 17240361 10.1016/j.biopsych.2006.10.006

[73] Nissen C, Holz J, Blechert J, Feige B, Riemann D, et al (2010) Learning as a model for neural plasticity in major depression. Biological psychiatry 68: 544–552. 20655508 10.1016/j.biopsych.2010.05.026

[74] Porsolt RD, Anton G, Blavet N, Jalfre M (1978) Behavioural despair in rats: a new model sensitive to antidepressant treatments. Eur J Pharmacol 47: 379–391. 204499 10.1016/0014-2999(78)90118-8

[75] Petit-Demouliere B, Chenu F, Bourin M (2005) Forced swimming test in mice: a review of antidepressant activity. Psychopharmacology (Berl) 177: 245–255. 15609067 10.1007/s00213-004-2048-7

[76] Nestler EJ, Hyman SE (2010) Animal models of neuropsychiatric disorders. Nature Neuroscience 13: 1161–1169. 20877280 10.1038/nn.2647 PMC3750731

[77] Sun P, Wang F, Wang L, Zhang Y, Yamamoto R, et al (2011) Increase in cortical pyramidal cell excitability accompanies depression-like behavior in mice: a transcranial magnetic stimulation study. J Neurosci 31: 16464–16472. 22072696 10.1523/JNEUROSCI.1542-11.2011 PMC6633240

[78] MacQueen GM, Ramakrishnan K, Ratnasingan R, Chen B, Young LT (2003) Desipramine treatment reduces the long-term behavioural and neurochemical sequelae of early-life maternal separation. Int J Neuropsychopharmacol 6: 391–396. 14641986 10.1017/S1461145703003729

[79] Aisa B, Tordera R, Lasheras B, Del Rio J, Ramirez MJ (2008) Effects of maternal separation on hypothalamic-pituitary-adrenal responses, cognition and vulnerability to stress in adult female rats. Neuroscience 154: 1218–1226. 18554808 10.1016/j.neuroscience.2008.05.011

[80] Romeo RD, Mueller A, Sisti HM, Ogawa S, McEwen BS, et al (2003) Anxiety and fear behaviors in adult male and female C57BL/6 mice are modulated by maternal separation. Hormones and behavior 43: 561–567. 12799173 10.1016/s0018-506x(03)00063-1

[81] Millstein RA, Holmes A (2007) Effects of repeated maternal separation on anxiety- and depression-related phenotypes in different mouse strains. Neurosci Biobehav Rev 31: 3–17. 16950513 10.1016/j.neubiorev.2006.05.003

[82] Kessler RC, Magee WJ (1993) Childhood adversities and adult depression: basic patterns of association in a US national survey. Psychol Med 23: 679–690. 8234575 10.1017/s0033291700025460

[83] Perona MT, Waters S, Hall FS, Sora I, Lesch KP, et al (2008) Animal models of depression in dopamine, serotonin, and norepinephrine transporter knockout mice: prominent effects of dopamine transporter deletions. Behav Pharmacol 19: 566–574. 18690111 10.1097/FBP.0b013e32830cd80f PMC2644662

